# Looking at Economics Through the Eyes of Thermodynamics: A New Approach to the Second Law of Economics, and Its Main Revelations

**DOI:** 10.3390/e28080846

**Published:** 2026-07-29

**Authors:** Vítor Costa

**Affiliations:** TEMA—Centro de Tecnologia Mecânica e Automação, Departamento de Engenharia Mecânica, Universidade de Aveiro, Campus Universitário de Santiago, 3810-193 Aveiro, Portugal; v.costa@ua.pt

**Keywords:** Thermodynamics–Economics analogy, Second Law of Economics, absolute merchandise economic temperature, absolute merchandise unit price, intrinsic demand curve

## Abstract

The Economics versions of the First and Second Laws of Thermodynamics are crucial for examining Economics through the lens of Thermodynamics. The First Law sets out conservation principles; the Second Law is required to describe what the First Law cannot: the one-way character of processes and the quantification of irreversibility (imperfection). A new approach is proposed to the Second Law of Economics, which includes as its main steps and results (*i*) a Second Law statement based on the observation that an isolated economic system evolves towards merchandise economic thermal equilibrium; (*ii*) the need to consider a new property, the merchandise economic entropy; (*iii*) a definition of the absolute merchandise economic temperature from the condition of maximum merchandise economic entropy for merchandise economic thermal equilibrium; (*iv*) the same sense of change in the merchandise economic temperature and in the number of traded merchandise units in an economic system; (*v*) an economic system has its intrinsic demand curve; (*vi*) a statement of the Third Law of Economics from the profile of the merchandise economic entropy function; (*vii*) the differential equation defining the merchandise economic entropy; and (*viii*) the definition of the merchandise economic entropy generation, the best indicator of the irreversibility (imperfection) of an economic process.

## 1. Introduction

Exploring analogies between Thermodynamics and other disciplines is not new. In particular, the Second Law of Thermodynamics has motivated many scientific and philosophical reflections and conjectures of several species in several domains. This is also the case when exploring analogies between Thermodynamics and Economics.

### 1.1. Previous Works

The first relevant work was published by Georgescu-Roegen, focusing on economic scarcity and long-term sustainability (impossibility of full recycling, due to material entropy increase) and thus on the one-way evolution direction set by the Second Law. Ref. [1] collected some of his most relevant publications, trying to set analogies between Mechanics, Thermodynamics, Production, and Economics. The work in ref. [2] is also a theoretical treatise, discussing evolution, entropy, order, probability, cause, purpose, evolution, value, and development. In ref. [3], connections between energy, entropy, Economics, evolution, ecology, and ethics are theoretically explored. The work of Georgescu-Roegen is still the seed for recent studies trying to relate Economics and Thermodynamics [4].

Bourley and Foster [5] edited a set of essays trying to establish analogies between the First and Second Laws of Thermodynamics and their Economics counterparts. The work of Schulz [6] is of a statistical nature, exploring similarities and parallelisms between Thermodynamics and Economics. Chen [7] and Kümmel [8] explore the analogies between Thermodynamics and Economics from a theoretical point of view.

Ayres and Nair [9] theoretically explore analogies between Thermodynamics and Economics, based on the following statement: ‘*The Laws of the conservation of energy and of the increase of entropy constrain the processes by which raw materials are transformed into consumable goods, and therefore have implications for the way economists model these processes*.’ In the work of Ayres [10], strong emphasis is placed on the Second Law’s one-way character, raising concerns about resources’ scarcity and sustainability. Saslow [11] proposed analogous variables and concepts for Economics as in Thermodynamics, without considering the dynamics of the economic processes and the one-way character of the Second Law. Belables [12] theoretically dissertated on the main lessons taught by Thermodynamics to economists and energy engineers.

All these works are, however, theoretical considerations that do not propose or set parallel structures for Economics and Thermodynamics, nor provide quantification tools for that.

In the work by Rozonoer [13], the author published a set of three papers in an attempt to reveal resource exchange and allocation mechanisms common for processes of a different nature (economic, sociological, physical, etc.). He described the equilibrium model based on the assumed existence of said ‘structural (extensive/additive) function’, interpreted as entropy in Thermodynamics and as utility in Economics. The economic and thermodynamic interpretations of basic concepts and relations involved in the model were given. Tsirlin and Gagarina [14] considered optimal trading processes in economic systems, basing the analysis on accounting for irreversibility factors using the wealth function and capital dissipation concepts. In a recent work, Jonath et al. [15] explored the Consumer-to-Producer Temperature Gradient; it is a ‘new leading economic indicator derived in relation to first principles and incorporates human nature into economic theory’. Bormashenko and Shendrik [16] proposed a redefinition of key notions in econophysics based on the Landauer principle. Marginal economic temperature was defined, economic entropy was redefined based on information processing, and the First and Third Laws of Economics–Thermodynamics were proposed. In the work by Zouari et al. [17], the Landauer principle is the motivation for the definition of economic temperature as the monetary price of processing one bit of information irreversibly, concluding that the thermodynamic framework provides a structure that standard-fee market analysis does not.

Even if these studies propose balance equations and inequalities that are the mathematical expressions of first principles, with some similarity to those considered and those emerging from the Economics–Thermodynamics analogy proposed by Costa [18,19,20], the involved economic analogs of temperature, energy, heat, work, entropy, and entropy generation (and thus, its consideration for quantification of economic irreversibility) have different meanings. The way to explore analogies between Thermodynamics and Economics, as proposed by Costa [18,19,20], is based on two main ideas:

- The number of merchandise units of an economic system is a property of the system. Similarly, the number of monetary units of an economic system is a property of the system. The number of (merchandise and monetary) units and their accounting is the basis of the Economics counterpart of the First Law’s balance equations.
- Merchandise is traded (transferred) in the increasing unit price direction, driven by a merchandise unit price difference (driven by a merchandise economic temperature difference), generating merchandise economic entropy (generating merchandise financial value). The one-way character of merchandise transfer in normal trading operations, from smaller to higher merchandise unit prices, and the merchandise financial value generation in trading operations is the Economics counterpart of the Second Law of Thermodynamics. Financial value generation is the Economics counterpart of entropy generation in Thermodynamics. Economic entropy accounting is the basis of the Economics counterpart of the Second Law’s balance equations.

A similar Four-Laws structure to Economics based on the well-established one to Thermodynamics has been proposed by Costa [19], which includes merchandise economic thermal equilibrium, merchandise economic temperature, the Zeroth Law of Economics, merchandise transfer interactions, merchandise wealth, merchandise unit generation, merchandise reservoir, monetary transfer interactions, monetary wealth, monetary units generation, merchandise and monetary exchanges in trading operations, (merchandise and monetary) units balance equations, the First Law of Economics, the Second Law of Economics, the Kelvin–Planck statement of the Second Law of Economics, the Clausius statement of the Second Law of Economics, economic Carnot cycle, economic reversibility and irreversibility, absolute merchandise economic temperature scale (absolute merchandise unit price scale), economic Bucher diagram, the Clausius inequality of Economics, merchandise economic entropy definition through a differential equation, economic entropy generation, economic entropy transfer interactions, economic entropy balance equations, merchandise economic entropy generation in merchandise transfer through a merchandise economic temperature difference, the Third Law of Economics, and the absolute value of the merchandise economic entropy.

The work by Costa [20] states, for the first time, the constitutive law setting how a change in the number of merchandise units of an economic system is related to the change in their merchandise economic temperature (in their merchandise unit price), crucial for the analysis of dynamic economic processes. The economic analog of exergy, the economic availability, is introduced for the first time, and the expression for its quantification is obtained.

The Second Law’s base statements can be different, even if many of the subsequent results are essentially the same. As this paper proposes a new approach to the Second Law of Economics when compared with the previously proposed one [18], a summary is made of the two different approaches. Even thus, it is noted that the new proposed approach reveals considerable and relevant information not revealed by the previous approach [19]. It is not claimed that one approach is better than the other; what is claimed is that these two different approaches have their own merits and that, considered together, they give a more complete picture of the Thermodynamics–Economics analogy, complementing and completing each other, without conflicting with each other but instead reinforcing each other.

### 1.2. Previous Approach to the Second Law of Economics, Following the One Based on Cyclically Operating Thermal Engines

The previous approach by Costa [19] closely followed that of many Engineering Thermodynamics textbooks [21], which, in turn, followed the historical developments of the Second Law of Thermodynamics. It starts with the observation that if two economic systems are allowed to exchange traded merchandise units, they exchange traded merchandise units up to a point when they cease that exchange. Under that condition, two economic systems are said to be in merchandise economic thermal equilibrium, and they share the same numerical value of a property, which is defined as the *empiric merchandise economic temperature* $\theta_{E,M}$ [U/€]. This allows for defining the property empiric merchandise economic temperature. Based on this, the *empiric merchandise unit price* $\pi_{M}$ [€/U] is defined as the inverse of the empiric merchandise economic temperature, that is, $\pi_{M}=1/\theta_{E,M}$ [€/U]. Based on this, the Zeroth Law of Economics is stated as the Law of Merchandise Economic Thermal Equilibrium.

Next, we define the number of merchandise and monetary units of an economic system, identify and quantify the merchandise and monetary units transfer interactions, and set the merchandise and monetary units balance equations for the economic system. These merchandise and monetary units balance equations correspond to the mathematical statement of the First Law of Economics.

Then, we note that some observations of economic activity reveal a one-way behavior, which the First Law of Economics is not able to describe (it results only in balance equations), which are the basis of the Second Law of Economics. These observations can be organized in the form of the Kelvin–Planck and Clausius statements of the Second Law of Economics. The concepts of economic reversibility and irreversibility, and of economic cycle, are also introduced.

The Kelvin–Planck statement of the Second Law of Economics allows us to set what is possible and what is impossible when an ‘economic engine’ operates cyclically when in contact with just one merchandise reservoir. The (reversible) economic Carnot cycle, operating when in contact with two merchandise reservoirs, allows us to define the lower limit for the ratio of traded merchandise transfer interactions involved in a cycle operating when in contact with two merchandise reservoirs and set the *absolute merchandise economic temperature* scale $T_{E,M}$ [U/€], based on the traded merchandise transfer interactions of the (reversible) economic Carnot cycle.

The next step consists of analyzing the economic cycle executed when in contact with any number of merchandise reservoirs, followed by what happens when the economic cycle is executed when in contact with an infinite number of merchandise reservoirs, thus leading to the Clausius inequality of Economics. The Clausius inequality of Economics, which is an equality for the reversible limit economic cycle, allows for obtaining the differential equation defining the merchandise economic entropy.

Any cycle can be considered to be composed of two processes, one reversible and the other arbitrary in terms of reversibility; the cyclic processes can be abandoned, and the result relative to an economic process executed when in contact with any number of merchandise reservoirs can thus be obtained. This allows us to define merchandise economic entropy generation as a measure of the economic irreversibility of an economic process.

This is the same sequence as that of Thermodynamics, based on the analysis and study of thermal engines operating cyclically to deliver mechanical work, in the search for their increased efficiency [21]. That led Clausius to introduce the (then) *new* property, *entropy*, coining the term ‘entropy’ (after the Greek word *ή* *τρ*o*πή*, meaning ‘transformation’, and as similar as possible to the word *energy*) and identifying entropy generation as ‘uncompensated heat’.

The previous steps are followed by identifying merchandise economic entropy as the financial value of the traded merchandise units and by identifying and quantifying the merchandise economic entropy transfer interactions. If the merchandise economic temperature is involved, it must be the absolute merchandise economic temperature. From that, we set the merchandise economic entropy balance equations.

The analysis of the traded merchandise transfer across a finite merchandise economic temperature difference, in the decreasing merchandise economic temperature direction (across a finite merchandise unit price difference, in the increasing merchandise unit price direction) as imposed by the Second Law of Economics, allows for identifying merchandise economic entropy generation as the profit generation (the financial value generation) in such a merchandise transfer (in such a trading operation).

After that, the Third Law of Economics is established, stating that an economic system whose merchandise units have a null merchandise economic temperature (have an infinite merchandise unit price) has a null merchandise economic entropy (has a null merchandise financial value).

### 1.3. New Proposed Approach to the Second Law of Economics, Following the One Based on the a Priori Introduced Merchandise Economic Entropy Property

The new proposed approach is different from the previous one, revealing different relations, results, and conclusions. It closely follows that of some Thermodynamics and Engineering Thermodynamics textbooks [22,23,24]. It starts by defining the number of merchandise and monetary units of an economic system, identifying and quantifying the merchandise and monetary transfer interactions, and setting the merchandise and monetary units balance equations, in the same way as the previous approach.

After that, it is observed that, in an isolated economic system, the evolution occurs in a well-defined direction, which the First Law (conservation principles) is unable to describe. This shows that a new property is required, the *merchandise economic entropy*, which is introduced as an extensive property of an economic system, and based on observations of economic processes, its characteristics and behavior are presented and discussed. Given its nature, it is seen that the merchandise economic entropy of an isolated economic system can only increase, up to a maximum when the economic system is in merchandise economic thermal equilibrium. This increasing condition is taken as the statement of the Second Law of Economics.

The condition of maximum merchandise economic entropy of an isolated economic system leads to the definition of the *absolute merchandise economic temperature*. In the previously proposed approach, the notion of merchandise economic thermal equilibrium leads to the statement of the Zeroth Law of Economics and to the definition of the *empiric* merchandise economic temperature. In the new approach, it is the condition of economic thermal equilibrium that allows the *absolute* merchandise economic temperature to be defined without the need to introduce the economic Carnot cycle.

The concavity of the function merchandise economic entropy of an isolated economic system allows us to set the monotonic variation in the number of merchandise units in the economic system and of the merchandise economic temperature of those merchandise units. The reduction in the number of merchandise units of the economic system is associated with the reduction in the merchandise economic temperature (is associated with the increase in the merchandise unit price) of the merchandise units in the economic system, and vice versa. This allows to state the Third Law of Economics for the limit of a null merchandise economic temperature (of an infinite merchandise unit price).

This monotonic behavior implies that the merchandise economic temperature (the merchandise unit price) of the merchandise units in the economic system does not depend just on the neighboring economic systems with which the economic system under analysis exchanges traded merchandise units (the scarcity or abundance of merchandise units in an economic system influences the merchandise economic temperature (the merchandise unit price) of these merchandise units) but that it is a characteristic of the economic system itself. This allows us to define the *intrinsic demand curve* of the economic system.

This sequence is different from the previously referred one in Section 1.2, as it starts from the observations of economic activity, the required existence of a property, the merchandise economic entropy, and its main characteristics and behavior. Of major relevance is the result stating that the merchandise economic entropy of an isolated economic system can only increase or, in the limit of no economic irreversibility *in* the economic system, maintain its value, this being taken as the base statement for the Second Law of Economics.

### 1.4. Main Results of Present Work

This work proposes a new approach to the Economics analog of the Second Law, whose main developments and steps are described in the previous Section 1.3. The main results are as follows:

- The statement of the Economics version of the Second Law based on the observation of an isolated economic system and on its evolution towards the merchandise economic thermal equilibrium (towards the merchandise unit price equilibrium).
- A new property, the *merchandise economic entropy*, is needed to describe what the First Law (conservation principles) is unable to describe.
- From the condition of merchandise economic thermal equilibrium, for which the merchandise economic entropy is maximum, the absolute merchandise economic temperature can be defined.
- From the condition of merchandise economic thermal equilibrium, for which the merchandise economic entropy is maximum, the absolute merchandise unit price can be defined.
- Based on the concavity of the merchandise economic entropy function, we can obtain the relevant result: if the merchandise economic temperature of the merchandise units *in* the economic system increases (if the merchandise unit price of the merchandise units *in* the economic system decreases), the number of merchandise units in the economic system tends to increase. Changing the order of the involved variables, if the number of merchandise units in the economic system increases, the merchandise economic temperature of the merchandise units in the economic system tends to increase (the merchandise unit price of the merchandise units in the economic system tends to decrease). This main result is also valid in the opposite direction, and the previous statements can be rewritten by changing ‘increase’ to ‘decrease’ and ‘decrease’ to ‘increase’.
- The previous result allows another relevant result to be obtained: an economic system has its own, and characteristic, *intrinsic demand curve*.
- Based on the intrinsic demand curve, it results that the merchandise demand is not only conditioned by the economic systems with which the economic system under analysis exchanges traded merchandise units but also conditioned by the economic system itself.
- From the profile of the variation in the merchandise economic entropy of the economic system with the number of traded merchandise units in the economic system, the statement of the Economics version of the Third Law is obtained: the merchandise economic entropy (the merchandise financial value) of the economic system whose merchandise economic temperature tends to zero (whose merchandise unit price tends to infinity) also tends to zero.
- From the conducted analyses and the obtained results, the differential equation defining the merchandise economic entropy is obtained.
- From the conducted analyses and the obtained results, the merchandise economic entropy generation is defined and the expression for its evaluation obtained, which can only be positive or, in the limit of economically reversible processes, null.
- From that, the merchandise and monetary economic entropy balance equations are obtained, similar to the ones obtained when following the previous approach.

### 1.5. Evolution Towards More Perfect (Less Irreversible) Economies

As referred to before by Costa [20], entropy generation is a measure of the imperfection of thermodynamic processes. Systems turned off, stopped, and not operating have no losses and no imperfections; the challenge of science and engineering is to have systems operating at moderate levels of entropy generation, which is the same as having systems operating at moderate levels of irreversibility (at moderate levels of imperfection) [21].

Similarly, economic entropy generation (financial value generation) can be considered a measure of the imperfection of economic processes. Economic systems not operating have no losses and no imperfections; the challenge is not frozen economies, but to have economic systems operating at moderate levels of economic entropy generation, which is the same as having economic systems operating at moderate levels of economic irreversibility (at moderate levels of economic imperfection). Looking at Economics through the eyes of Thermodynamics searches for more perfect (less irreversible) economic processes and for opportunities to deliver more merchandise wealth, taken as the major objective of the economic processes, instead of the maximization of economic entropy generation (instead of the maximization of financial value generation).

The presented developments and proposed approaches challenge the ways of thinking, the reading and interpreting of the messages they contain, and how they are related to the real-world economy, the concepts, the paradigms, and the ways of thinking and acting toward more efficient economies. This gives new insights and opens up new ways of creating more perfect and more efficient economies, as well as methods of Economics thinking, teaching, and research.

The approach to set the proposed Thermodynamics–Economics analogy follows an essentially empirical method, based on observations from the economic activity. It starts by defining the number of merchandise and monetary units of an economic system as properties of the economic system and stating their balance equations. This is empirical knowledge resulting from observation. The one-way behavior of Economics, the economic activity developing in the direction of financial value generation, is also empirical knowledge resulting from observation. However, as the setting of the proposed analogy has Thermodynamics, a well-established area, behind it, which can be based on statements derived from empirical observation or based on an axiomatic framework, some ‘contamination’ by the axiomatic Thermodynamics framework exists of the followed empirical approach.

## 2. More Relevant Previous Developments and Results

This section summarizes the main concepts, developments, and results of previous works by Costa [18,19,20,25], which must be seen as relevant for the new proposed approach. The content of Section 2.1 and Section 2.2 closely follows that of Sections 2.1 and 2.3 of Costa [20].

### 2.1. Similar Variables and Terms

Table 1 summarizes the analogy between Thermodynamics and Economics as proposed by Costa [18,19], as condensed in Section 2 of Costa [20].

Only one merchandise species is considered; if more than one merchandise species is involved, additive contributions of all the merchandise species need to be considered. Units are indicated inside square brackets for all variables, equations, and inequations, for increased clarity, accuracy, and easier reading and understanding. Euro (€) is used as the monetary unit; however, other monetary units can be equally considered.

Of special relevance is the concept of merchandise wealth as merchandise that is not in the market and that thus has an infinite economic temperature (a null unit price) as the analog of mechanical work in Thermodynamics, which can be seen as heat at infinite temperature.

### 2.2. Merchandise Unit Balance Equation

An equation similar to the energy balance equation can be written, setting the balance of the number of merchandise units in an economic system. Written in the same form as usual in Engineering Thermodynamics [26], on a time rate basis, it states that

time rate of changeof the number ofmerchandise unitsin the system︸in the system=net flow rate oftraded merchandiseunits entering thesystem−net flow rate ofwealth (non-traded)merchandise unitsleaving the system︸through the system's boundary+ net generationrate of merchandiseunits in the system︸in the system [U/s]

Using the involved variables, such an equation can be written as

(1) $$\frac{dN_{M}}{dt}=\sum_{j=1}^{N_{Mj}}{\dot{M}}_{j}-{\dot{W}}_{M}+{\dot{N}}_{M,g}\;[U/s]$$

The traded merchandise transfer interactions ${\dot{M}}_{j}$ [U/s] are positive when entering the economic system and negative otherwise. The merchandise wealth transfer interactions are taken as positive when released by the economic system, assuming that the positive effect of the *economic engine* is to deliver merchandise wealth, similarly to that in Thermodynamics where the delivered mechanical work is the positive effect of the thermal engine [26].

Multiplying Equation (1) by *dt* leads to the differential form of the merchandise unit balance equation:

(2) $$dN_{M}=\sum_{j=1}^{N_{Mj}}\delta M-\delta W_{M}+\delta N_{M,g}\;[U]$$

Symbol d is used for the differential of an exact differential (a property of the system); $\int_{1}^{2}d\left(\cdot \right)={\left(\cdot \right)}_{2}-{\left(\cdot \right)}_{1}$, $\oint d\left(\cdot \right)=0$, and ${\left(\cdot \right)}_{1}$, and ${\left(\cdot \right)}_{2}$ have physical meanings; symbol δ is used for the differential of an inexact differential (a non-property of the system, not characterizing one state of the system, but instead a transfer interaction, referring to a process); $\int_{1}^{2}\delta \left(\cdot \right)\neq {\left(\cdot \right)}_{2}-{\left(\cdot \right)}_{1}$, $\oint \delta \left(\cdot \right)\neq 0$, and ${\left(\cdot \right)}_{1}$, and ${\left(\cdot \right)}_{2}$ have no physical meanings [26,27].

Energy is conserved. On the contrary, merchandise units can be generated or destroyed *in* the economic system [18,19,20]. *New* (additional) numbers of merchandise units can be generated in the economic system, cases for which ${\dot{N}}_{M,g}\geq 0$ [U/s]; when merchandise units are destroyed in the economic system, ${\dot{N}}_{M,g}\leq 0$ [U/s].

Monetary units balance equations can be similarly set [18,19,20]; however, they are not included here as they are not relevant to the purposes of this paper.

### 2.3. One Notable Difference Between Thermodynamics and Economics

It is important to note one main difference between Thermodynamics and Economics. When a thermodynamic system is subject to energy transfer interactions, the energy conservation principle (First Law) imposes how its internal energy changes due to these energy transfer interactions. As the internal energy and temperature levels of a thermodynamic system are related in some way [21], energy transfer interactions, internal energy change, and temperature change are related. If we transfer the energy of 5000 J as heat to 1 kg of water initially at 20 °C, the final temperature of the water is not arbitrary. From the energy balance equation, the change in the internal energy of the water due to the heat it receives is determined, and thus so is the internal energy of the water at the end of the heating process. As the internal energy of the water and its temperature are related through well-established relationships, the final temperature of the water is thus well determined.

This is not the case in Economics, as there is no direct relation between the merchandise units transfer interactions and the merchandise economic temperature change. When compared with Thermodynamics, this represents an area open for arbitrary decisions. The main consequence is that there is no closed relationship between the merchandise transfer interactions of an economic system and the merchandise economic temperature change, which is an arbitrary decision of the economic system controller (however, always searching for profit (financial value generation)). If we transfer 5000 U of merchandise units to an economic system, the merchandise unit price of the merchandise units initially in the system being equal to 3 €/U, the merchandise unit price of the final merchandise units in the system is arbitrary. As there is no closed relationship between the number of merchandise units in the system and their merchandise economic temperature, the change suffered by the merchandise economic temperature in the process cannot be obtained. It is the economic system controller who decides the unit price of the merchandise units in the economic system at the end of the process, however, and as previously referred to, always searching for profit (financial value generation). For example, if he/she purchases the 5000 merchandise units at a unit price of 2.8 €/U, he/she can decide to give at least a unit price of 3 €/U to all merchandise units in the economic system at the end of the process. In what concerns the *new* 5000 U merchandise units that entered the economic system, he/she expects to obtain at least 5000 [U] × (3.0 − 2.8) [€/U] = 1000 € profit (financial value generation).

## 3. New Proposed Approach and Its Main Results

The main steps of the new proposed approach are described in Section 1.3; the corresponding developments, details, analyses, discussions, and results are the content of this section.

### 3.1. Relevant Observation from a Thermodynamic System

We consider first the (thermodynamic) situation of a closed and isolated system, composed of $m_{j}$ [kg], j=1,2,…,N, masses of incompressible materials, each with its own (constant) specific heat capacity $c_{j}$ [J/(kg.K)], and each mass $m_{j}$ [kg] being initially at the respective temperature $T_{j}$ [K]. The total mass of the closed system is [21]

(3) $$m=\sum_{j=1}^{N}m_{j}\;\;[kg]$$

and its total internal energy is

(4) $$U_{i}=\sum_{j=1}^{N}U_{j}=\sum_{j=1}^{N}{\left(mc\right)}_{j}\left(T_{j}-T_{ref}\right)\;\;[J]$$

where $T_{ref}$ [K] is a reference temperature. As the thermodynamic system under analysis is isolated, mass m [kg] and internal energy U [J] remain constant, no matter the transformations and processes it suffers.

If masses $m_{j}$ [kg], j=1,2,…,N, are allowed to exchange energy as heat between them, with heat transfer being driven (motivated) by the temperature differences between them, heat flowing spontaneously from higher temperatures to lower temperatures, after a sufficiently long time, the transfer of energy as heat in the system ceases. All masses are in a thermal equilibrium state in that limit, a situation when they are all at the same temperature $T_{f}$ [K]. There are well-established physical constitutive laws describing and quantifying the heat exchanges between two bodies at different temperatures, with heat flowing in the decreasing temperature direction [28]. In the final situation of thermal equilibrium, the internal energy of the system can be evaluated as

(5) $$U_{f}=\sum_{j=1}^{N}{\left(mc\right)}_{j}\left(T_{f}-T_{ref}\right)=\left(T_{f}-T_{ref}\right)\sum_{j=1}^{N}{\left(mc\right)}_{j}\;\;[J]$$

which is numerically equal to the one given by Equation (4). From Equations (4) and (5), it is obtained that the final temperature $T_{f}$ [K] is

(6) $$T_{f}=\left[\sum_{j=1}^{N}{\left(mc\right)}_{j}T_{j}\right]/\left[\sum_{j=1}^{N}{\left(mc\right)}_{j}\right]\;\;[K]$$

It is well-known that what is observed is the *natural* evolution of the masses from their initial states at different temperatures to the final thermal equilibrium state, when all masses are at the same temperature (final thermal equilibrium) and not the inverse one, starting from all masses at the same temperature $T_{f}$ [K] and ending with the masses $m_{j}$ [kg], each at its own temperature $T_{j}$ [K], different from mass to mass. It is thus clear that there is something that cannot be described by the First Law of Thermodynamics (energy conservation), as energy is conserved in both considered processes, changing from $U_{i}$ [J] to $U_{f}=U_{i}$ [J] in the first one and from $U_{f}$ [J] to $U_{i}=U_{f}$ [J] in the second one. From the First Law’s viewpoint (energy conservation), both processes are equally possible.

This observation motivated the introduction of property entropy, S [J/K], an extensive property (proportional to the size of the system, quantified by its mass m [kg]), with the Second Law statement being based on the observation that the entropy of an isolated system cannot decrease, that is, the possible processes experienced by the isolated system are the ones for which

(7) $${\left(S_{f}\geq S_{i}\right)}_{\begin{array}{l} \mathrm{isolated}\\ \mathrm{system} \end{array}}\;\;[J/K]$$

and the processes for which ${\left(S_{f}<S_{i}\right)}_{\begin{array}{l} \mathrm{isolated}\\ \mathrm{system} \end{array}}$ [J/K] are impossible. However, for many, it is difficult to understand where the entropy increase (the entropy generation) comes from if the mass and energy remain constant in the system.

If there is some entropy increase, that means that there is some irreversibility in the process undergone by the system, and the inequality sign prevails in Equation (7). On the contrary, the equality sign prevails in Equation (7) if the process undergone by the system is reversible, that is, the process undergone by the system can be *reversed* with no need for any additional effects.

### 3.2. Relevant Observation from an Economic System

We now consider the isolated economic system composed of $N_{M,j}$ [U], j=1,2,…,N, numbers of merchandise units, each initially at the respective merchandise economic temperature $T_{E,M,j}$ [U/€] (each initially at the respective merchandise unit price $p_{M,j}$ [€/U]). The isolated economic system can be a village, a county, a city, etc. The total number of merchandise units of the isolated economic system is

(8) $$N_{M}=\sum_{j=1}^{N}N_{M,j}\;\;[U]$$

As the economic system under analysis is isolated, its number of merchandise units $N_{M}$ [U] remains constant, no matter the economic transformations and processes it suffers, under the condition that no merchandise units generation occurs in the system, that is, under the condition that $N_{M,g}=0$ [U], and that there are no merchandise wealth units in the economic system, that is, $N_{M,W}=0$ [U]. It is noted that in the economic system, $N_{M}=N_{M,M}+N_{M,W}$ [U] merchandise units of a given species can exist, where $N_{M,M}$ [U] is the number of tradable merchandise units and $N_{M,W}$ [U] is the number of merchandise wealth units (merchandise units set aside, not to be traded). The financial value of the merchandise units in the system in this initial state is evaluated as

(9) $$V_{f,i}=\sum_{j=1}^{N}N_{M,j}\cdot p_{M,j}\;\;[€]$$

If merchandise units are exchanged in normal trading operations in the isolated economic system, from seller to buyer, driven (motivated) by merchandise economic temperature differences (driven by merchandise unit price differences), traded merchandise *flowing spontaneously* from higher merchandise economic temperatures to lower merchandise economic temperatures (*flowing spontaneously* from lower merchandise unit prices to higher merchandise unit prices), after a sufficiently long time, the transfer (trading) of merchandise units in the economic system ceases, and they are in merchandise economic thermal equilibrium when they are all ($N_{M}$ [U]) at the same merchandise economic temperature $T_{E,M,f}$ [U/€] (when they are all at the same merchandise unit price $p_{M,f}$ [U/€]).

Contrary to what happens in heat transfer, there are no well-established constitutive law describing and quantifying the traded merchandise transfer between two economic systems at different merchandise economic temperatures. It is interesting to note, however, that even if there are no physical laws for that, there are laws imposed by governments and committees, stating that it is forbidden to sell a merchandise at a lower price than that at which it has been purchased, thus imposing by law that the merchandise transfer in normal trading operations occurs in the increasing merchandise unit price direction (occurs in the decreasing merchandise economic temperature direction).

When 1 kg of beans is purchased at the unit price of 1.2 €/kg and is sold at the unit price of 1.5 €/kg, the profit generation is 0.3 €/kg of beans traded in the system. No beans in the economic system are ‘wealth beans’, as $N_{M,W}=0$ [U]. Maybe some services need to be contracted to conduct the trading operation (labor, lighting, communications, cleaning services, etc.), and the effective profit resulting from 1 kg of beans traded is not equal to 0.3 €/kg. However, for our purposes, what is relevant is the increase in financial value (the financial value generation) by each traded kg of beans. No *motivation* exists for beans trading if there is no profit generation.

The financial value of the merchandise units in the economic system in the final merchandise economic thermal equilibrium state is evaluated as

(10) $$V_{f,f}=\left(\sum_{j=1}^{N}N_{M,j}\right)p_{M,f}\;\;[€]$$

obeying

(11) $$V_{f,f}=\left(\sum_{j=1}^{N}N_{M,j}\right)p_{M,f}\geq \sum_{j=1}^{N}N_{M,j}\cdot p_{M,j}=V_{f,i}\;\;[€]$$

and it is thus

(12) $$p_{M,f}\geq \left(\sum_{j=1}^{N}N_{M,j}\cdot p_{M,j}\right)/\left(\sum_{j=1}^{N}N_{M,j}\right)\;\;[€]$$

It is well known from observation of the economic activity that what happens is the *natural* evolution of the economic system from the initial state (each number of merchandise units $N_{M,j}$ [U] at its own merchandise economic temperature $T_{E,M,j}$ [U/€] (at its own merchandise unit price $p_{M,j}$ [€/U])) to the final equilibrium state when all the merchandise units are at the same merchandise economic temperature $T_{E,M,f}$ [U/€] (when they are all at the same merchandise unit price $p_{M,f}$ [U/€]), and not the inverse one, starting from all the merchandise units $N_{M}$ [U] at the same merchandise economic temperature $T_{E,M,f}$ [U/€] and ending with the numbers of merchandise units $N_{M,j}$ [U] j=1,2,…,N, each at its own merchandise economic temperature $T_{E,M,j}$ [U/€] (each at its own merchandise unit price $p_{M,j}$ [U/€]), different for each *j*. It is thus clear that there is something that cannot be described by the First Law of Economics (number of merchandise units conserved), as the number of merchandise units is conserved in both considered processes.

This observation motivates the introduction of the property *merchandise economic entropy*, $S_{E,M}$ [€], an extensive property (proportional to the size of the system, quantified by its number of merchandise units), with the economic Second Law statement stating that the merchandise economic entropy of an isolated economic system cannot decrease, that is, the possible processes experienced by the isolated economic system are the ones for which

(13) $${\left(S_{E,M,f}\geq S_{E,M,i}\right)}_{\begin{array}{l} \mathrm{isolated}\;\mathrm{economic}\\ \mathrm{system},\;N_{M,g}=N_{M,W}=0 \end{array}}\;\;[€]$$

and the processes for which ${\left(S_{E,M,f}<S_{E,M,i}\right)}_{\begin{array}{l} \mathrm{isolated}\;\mathrm{economic}\\ \mathrm{system},\;N_{M,g}=N_{M,W}=0 \end{array}}$ [€] are impossible. However, the same main question arises as in Thermodynamics: where does the merchandise economic entropy increase (the merchandise economic entropy generation) come from if the number of merchandise units remains constant in the economic system?

If there is some merchandise economic entropy increase, that means that there is some economic irreversibility in the process undergone by the economic system, and the inequality sign prevails in Equation (13). On the contrary, the equality sign prevails in Equation (13) if the process undergone by the economic system is reversible, that is, if the process undergone by the economic system can be *reversed* with no need for any additional effects.

### 3.3. On the Property Merchandise Economic Entropy

Merchandise economic entropy is an extensive property of an economic system, proportional to the economic system’s size (proportional to the number of traded merchandise units ($N_{M,W}=0$ [U]) in the economic system). Thus, the merchandise economic entropy of an economic system composed of several parts equals the sum of the merchandise economic entropy of the parts that compose the whole system. If the economic system $\left(C\right)$ is composed of parts $\left(A\right)$ and $\left(B\right)$, the merchandise economic entropy of the composite system $\left(C\right)=\left(A\right)+\left(B\right)$ is

(14) $$S_{E,M,C}=S_{E,M,A}+S_{E,M,B}\;\;[€]$$

The merchandise economic entropy is not a conserved property, and contrary to the number of merchandise units $N_{M}$ [U], it does not obey a conservation principle. As observed before, it must increase when economic spontaneous processes occur in an isolated economic system under the referred conditions.

From previous analysis and developments, the merchandise economic entropy must be equal to, or proportional to, the financial value of the tradable merchandise units in the economic system, and its units must be [€].

### 3.4. The Second Law of Economics Base Statement

The Second Law of Economics can be stated through the condition expressed by Equation (13), stating that the merchandise economic entropy of an isolated economic system (where no generation or destruction of merchandise units occurs and no merchandise wealth units exist) can only increase or, in the reversible limit, remain constant. This statement can be written in the differential form as

(15) $${\left(dS_{E,M}\right)}_{\begin{array}{l} \mathrm{isolated}\;\mathrm{economic}\\ \mathrm{system},\;\delta N_{M,g}=N_{M,W}=0 \end{array}}\geq 0\;\;[€]$$

### 3.5. Defining the Absolute Merchandise Economic Temperature

**Figure 1:**
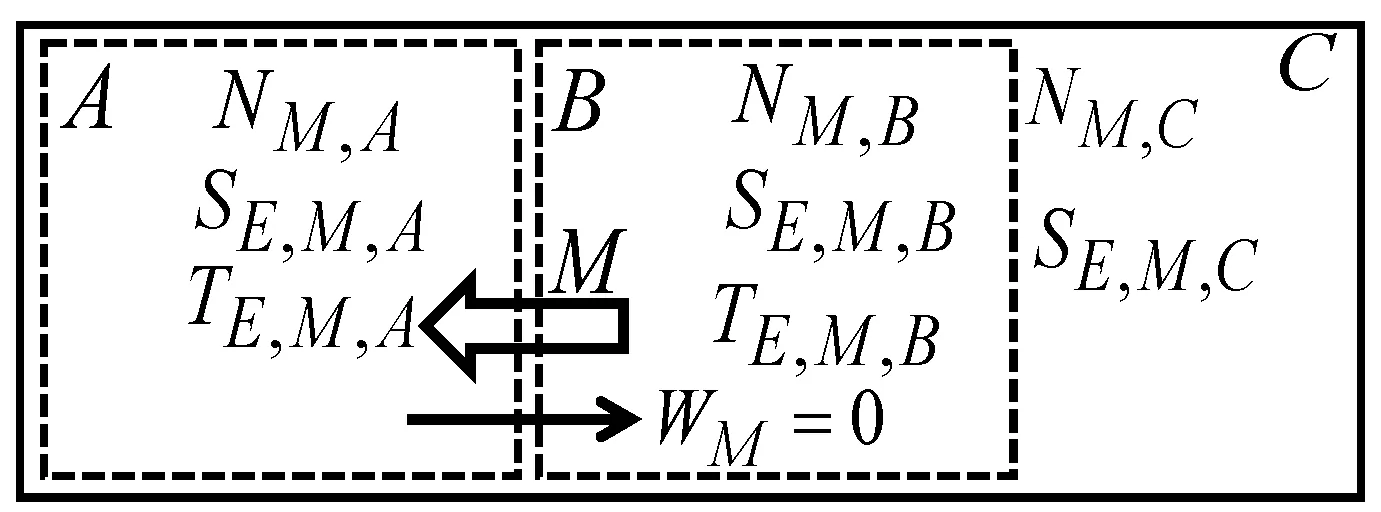
Schematic representation of the isolated economic system *C* as composed of systems *A* and *B*, which can only exchange traded merchandise units but not merchandise wealth units.

Consider two economic systems *A* and *B*, which are each in merchandise economic thermal equilibrium, even if systems *A* and *B* are not in merchandise economic thermal equilibrium with each other. Initially, system *A* has $N_{M,A}$ [U] merchandise units at the merchandise economic temperature $T_{E,M,A}$ [U/€], and system *B* has $N_{M,B}$ [U] merchandise units at the merchandise economic temperature $T_{E,M,B}$ [U/€]. Systems *A* and *B* can only exchange merchandise units as traded merchandise units M [U], but not as merchandise wealth $\left(W_{M}=0\right)$ [U]. Systems *A* and *B* form the isolated economic system *C*, for which, as illustrated in Figure 1,

(16) $$N_{M,C}=N_{M}=N_{M,A}+N_{M,B}=\mathrm{constant}\;\;[U]$$

and defining the dimensionless number of merchandise unit repartition ratio *r* as

(17) $$r=N_{M,A}/N_{M}\;\;[-]$$

it is

(18a) $$N_{M,A}=rN_{M}\;\;[U]$$

(18b) $$N_{M,B}=\left(1-r\right)N_{M}\;\;[U]$$

As merchandise economic entropy $S_{E,M}$ [€] is an extensive property, the merchandise economic entropy of system *C*, composed of systems *A* and *B*, is given by Equation (14), where $S_{E,M,A}=S_{E,M,A}\left(N_{M,A}\right)$ [€] and $S_{E,M,B}=S_{E,M,B}\left(N_{M,B}\right)$ [€]. However, contrary to what happens with the numbers of merchandise units of systems *A* and *B*, which obey Equation (16), no similar equation can be derived stating the merchandise economic entropy of system *C* to be constant.

**Figure 2:**
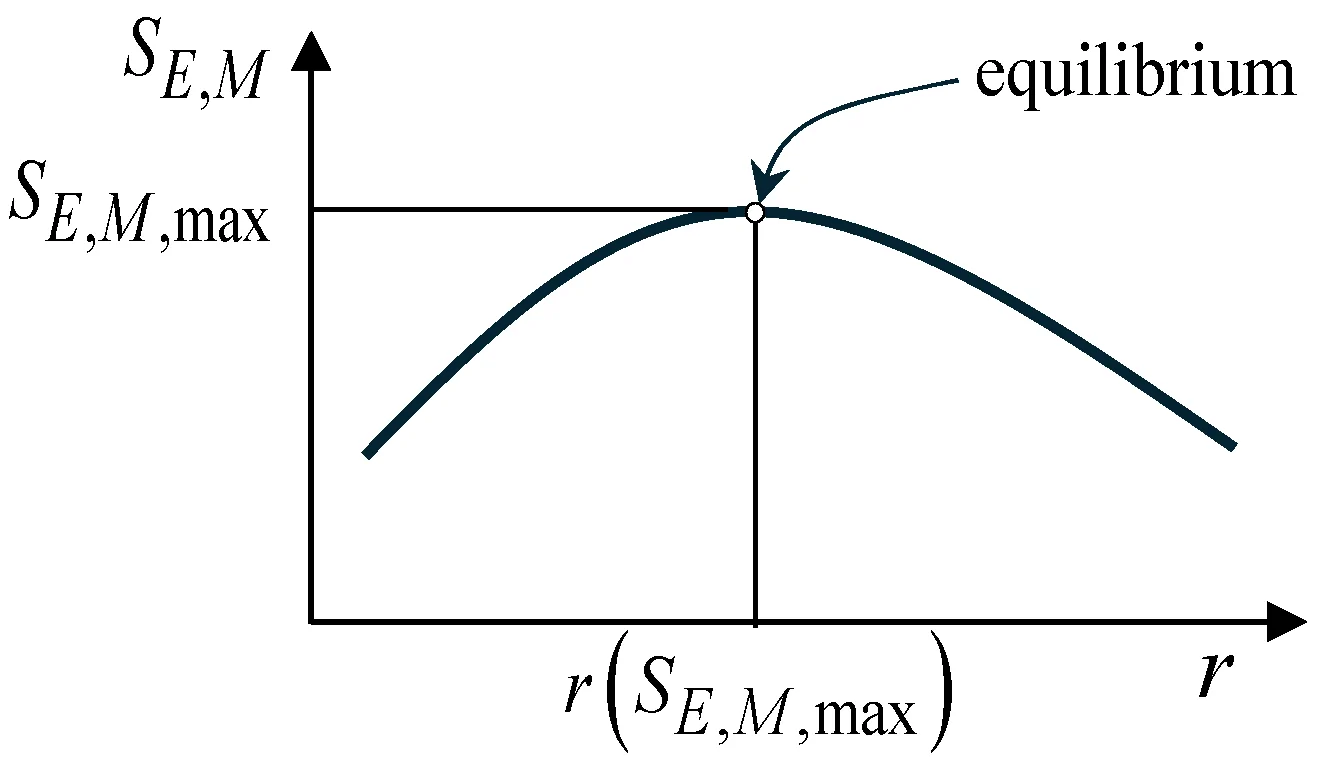
Schematic representation of the maximum merchandise economic entropy of isolated economic system *C* composed of systems *A* and *B* when they are in merchandise economic thermal equilibrium.

As economic systems *A* and *B* are allowed to exchange traded merchandise units between them, which happens *spontaneously* only if the merchandise economic entropy of the composite economic system *C* increases, in the limit when economic systems *A* and *B* remain in economic contact but cease exchanging traded merchandise units, they are in merchandise economic thermal equilibrium. Thus, in the merchandise economic thermal equilibrium condition, systems *A* and *B* are each in merchandise economic thermal equilibrium, and additionally, they are in mutual merchandise economic thermal equilibrium, a situation which corresponds to the maximum merchandise economic entropy of composite system *C*. Under the merchandise economic thermal equilibrium condition, systems *A* and *B* share the same value of merchandise economic temperature. This is schematically illustrated in Figure 2.

Taking Figure 2 into consideration, in the merchandise economic thermal equilibrium state, we have

(19) $$\frac{d\left(S_{E,M,C}\right)}{dr}=0\;\;[€]$$

Using Equation (14) and the chain derivative rule,

(20) $$\frac{d\left(S_{E,M,C}\right)}{dr}={\left[\left(\frac{\partial S_{E,M,A}}{\partial N_{M,A}}\right)\frac{dN_{M,A}}{dr}+\left(\frac{\partial S_{E,M,B}}{\partial N_{M,B}}\right)\frac{dN_{M,B}}{dr}\right]}_{W_{M}=N_{M,g}=N_{M,W}=0}\;\;[€]$$

which, considering Equations (18a) and (18b), can be written as

(21) $$\frac{d\left(S_{E,M,C}\right)}{dr}={\left[\left(\frac{\partial S_{E,M,A}}{\partial N_{M,A}}\right)-\left(\frac{\partial S_{E,M,B}}{\partial N_{M,B}}\right)\right]}_{W_{M}=N_{M,g}=N_{M,W}=0}N_{M}\;\;[€]$$

and as in the merchandise economic thermal equilibrium conditions, $d\left(S_{E,M,C}\right)/dr=0$ [€], it is thus

(22) $${\left[\left(\frac{\partial S_{E,M,A}}{\partial N_{M,A}}\right)=\left(\frac{\partial S_{E,M,B}}{\partial N_{M,B}}\right)\right]}_{\begin{array}{l} W_{M}=N_{M,g}=N_{M,W}=0\\ \mathrm{merchandise}\;\mathrm{economic}\\ \mathrm{thermal}\;\mathrm{equilibrium} \end{array}}\;\;[€/U]$$

The left-hand side of Equation (22) is a property of system *A*, and the right-hand side of Equation (22) is a property of system *B*. Under the merchandise economic thermal equilibrium condition, systems *A* and *B* share the same value of merchandise economic temperature, which can thus be defined as any function of ${\left(\partial S_{E,M}/\partial N_{M}\right)}_{\delta W_{M}=\delta N_{M,g}=N_{M,W}=0}$ [€/U]. One of the simplest alternatives, chosen such that the traded merchandise units flow *spontaneously* in the decreasing economic temperature direction, is to define the absolute merchandise economic temperature as

(23) $$T_{E,M}\equiv {\left[{\left(\frac{\partial S_{E,M}}{\partial N_{M}}\right)}^{-1}\right]}_{\begin{array}{l} \delta W_{M}=\delta N_{M,g}=N_{M,W}=0\\ \mathrm{merchandise}\;\mathrm{economic}\\ \mathrm{thermal}\;\mathrm{equilibrium} \end{array}}\;\;[U/€]$$

such that Equation (22) can be rewritten as

(24) $${\left[T_{E,M,A}={\left(\frac{\partial S_{E,M,A}}{\partial N_{M,A}}\right)}^{-1}=T_{E,M,B}={\left(\frac{\partial S_{E,M,B}}{\partial N_{M,B}}\right)}^{-1}\right]}_{\begin{array}{l} \delta W_{M}=\delta N_{M,g}=N_{M,W}=0\\ \mathrm{merchandise}\;\mathrm{economic}\\ \mathrm{thermal}\;\mathrm{equilibrium} \end{array}}\;\;[U/€]$$

Previous results highlight that the merchandise economic temperature can be defined only under merchandise economic thermal equilibrium conditions (the merchandise economic temperature can be defined only for stable equilibrium states). If an economic system is not under merchandise economic thermal equilibrium, it is in a metastable state for which the merchandise economic temperature is not defined.

In the previous equations, it must be retained that $T_{E,M}$ [U/€] is a property and that the derivatives are derivatives of properties, which refer to a state, but that $\delta W_{M}$ [U] and $\delta N_{M,g}$ [U] refer to processes. This is not a problem, as the derivative $\left(\partial S_{E,M}/\partial N_{M}\right)$ [€/U] can be understood as involving the infinitesimal change in variables $S_{E,M}$ [€] and $N_{M}$ [U] in an infinitesimal process in the vicinity of that state, for null merchandise wealth transfer interactions and null merchandise units generation in that process, that is, for $\delta W_{M}=\delta N_{M,g}=0$ [U].

It is noted that as $S_{E,M}$ [€] and $N_{M}$ [U] are extensive properties, the so-defined merchandise economic temperature $T_{E,M}$ [U/€] is an intensive property (not proportional to the economic system size, expressed by its number of tradable merchandise units).

If economic systems *A* and *B* are each in merchandise economic thermal equilibrium even if they are not in mutual economic thermal equilibrium, using Equation (23), Equation (21) can be written as

(25) $$d\left(S_{E,M,C}\right)=\left(N_{M}\right)dr\left(\frac{1}{T_{E,M,A}}-\frac{1}{T_{E,M,B}}\right)\;[€]$$

As explained before, we forcibly have $d\left(S_{E,M,C}\right)\geq 0$ [€], and if $T_{E,M,B}>T_{E,M,A}$ [U/€], it is $\left(1/T_{E,M,A}-1/T_{E,M,B}\right)>0$ [€/U], thus requiring dr>0 [−], that is, from Equations (18a) and (18b), $N_{M,A}$ [U] increases and $N_{M,B}$ [U] decreases when economic systems *A* and *B* spontaneously exchange traded merchandise units and the combined system *C* evolves towards merchandise economic thermal equilibrium. This requires the spontaneous traded merchandise units transfer from system *B* to system *A*, thus, requiring the spontaneous traded merchandise units transfer in the decreasing merchandise economic temperature direction, from economic system *B* at merchandise economic temperature $T_{E,M,B}$ [U/€] to economic system *A* at merchandise economic temperature $T_{E,M,B}$ [U/€], for $T_{E,M,B}>T_{E,M,A}$ [U/€].

A different result could be obtained if the economic temperature definition is other than that set by Equation (23), always obeying Equation (22). For example, if the absolute merchandise unit price is defined from Equation (24) as

(26) $$p_{M}\equiv \frac{1}{T_{E,M}}={\left[\left(\frac{\partial S_{E,M}}{\partial N_{M}}\right)\right]}_{\begin{array}{l} \delta W_{M}=\delta N_{M,g}=N_{M,W}=0\\ \mathrm{merchandise}\;\mathrm{economic}\\ \mathrm{thermal}\;\mathrm{equilibrium} \end{array}}\;\;[€/U]$$

Equation (25), written as

(27) $$d\left(S_{E,M,C}\right)=\left(N_{M}\right)dr\left(p_{M,A}-p_{M,B}\right)\;\;[€]$$

requires the traded merchandise transfer in the increasing merchandise unit price direction, from $p_{M,B}$ [€/U] to $p_{M,A}$ [€/U], for $p_{M,B}<p_{M,A}$ [€/U].

At this point, it is important to note that the absolute merchandise economic temperature $T_{E,M}$ [U/€] as defined by Equation (23), the traded merchandise transfers occurring in the decreasing merchandise economic temperature direction, can be adopted or that the absolute merchandise unit price $p_{M}$ [€/U] as defined by Equation (26), the traded merchandise transfers occurring in the increasing merchandise unit price direction, can be adopted. Society and economies adopted the merchandise unit price, $p_{M}$ [€/U], with traded merchandise transfers occurring in the increasing merchandise unit price direction, and not its inverse, the merchandise economic temperature $T_{E,M}$ [U/€], and gave economic entropy (the financial value) the unit [€]. In accordance with that, when merchandise unit prices are high, merchandise economic temperatures are low, and the economy must be referred to as *cold*; on the contrary, when merchandise unit prices are low, merchandise economic temperatures are high, and the economy must be referred to as *hot*. This is not the language usually used, as it does not consider the inverse relationship between the merchandise economic temperature and the merchandise unit price. If the unit [thermium] had been given to the absolute merchandise economic temperature, the merchandise economic entropy property would have units of [U/thermium], the inverse of the absolute merchandise economic temperature (the absolute merchandise unit price) would have units of [1/thermium], and the unit of absolute merchandise economic temperature [thermium] would be equivalent to [U/€]. Similarly, the absolute temperature T [K] can be adopted; heat transfers occur in the decreasing temperature direction, or the inverse of the absolute temperature 1/T [1/K] can be adopted, with heat transfers occurring in the increasing 1/T [1/K] direction. Thermodynamics adopted the absolute temperature T [K], heat transfers occurring in the decreasing temperature direction, and gave it the unit [K], not the inverse of the absolute temperature 1/T [1/K]. Curiously, in Thermodynamics, there are many instances in which the absolute temperature T [K] is not involved but instead, the inverse of the absolute temperature, 1/T [1/K], is [21,26]. If the unit [entropium] had been given to entropy, the absolute temperature (defined from the entropy property through an equation analog to Equation (25)) would have units of [J/entropium], the inverse of the absolute temperature would have units of [entropium/J], and the unit of absolute temperature [K] would be equivalent to [J/entropium].

The result of Equation (26) must be carefully interpreted. If it is $\delta W_{M}=\delta N_{M,g}=N_{M,W}=0$ [U], the unique $N_{M}$ [U] merchandise units in the system are tradable merchandise units, and under the conditions from Equation (2), it is $\delta M=dN_{M}$ [U]. If the system is in merchandise economic thermal equilibrium at the merchandise unit price $p_{M}$ [€/U] (at the merchandise economic temperature $T_{E,M}=1/p_{M}$ [U/€]) and if it exchanges (releases or receives) $\delta M=dN_{M}$ [U] traded merchandise units which cross the economic system boundary at the merchandise economic temperature $T_{E,M}=1/p_{M}$ [U/€] (at the merchandise unit price $p_{M}$ [€/U]), the change in the merchandise economic entropy (the change in the merchandise financial value) of the economic system is $dS_{E,M}=\delta M\cdot p_{M}=dN_{M}\cdot p_{M}$ [€]. It is $dS_{E,M}>0$ [€] if $\delta M=dN_{M}>0$ [U] (if traded merchandise units enter the economic system), and it is $dS_{E,M}<0$ [€] if $\delta M=dN_{M}<0$ [U] (if traded merchandise units leave the economic system). It is thus clear that the meaning of the absolute merchandise unit price as defined by Equation (26) is the same as the usual meaning of the merchandise unit price.

Previous developments considered partial derivatives of $S_{E,M,A}$ [€] and $S_{E,M,B}$ [€], as from Thermodynamics, it is well known that entropy depends on two other properties, in this case, used as the base of the proposed analogy internal energy *U* [J] and volume *V* [m^3^] [23], and it is thus $S=S\left(U,V\right)$ [J/K]. The developments in [23] consider the partial derivative ${\left(\partial S/\partial U\right)}_{V}$ [1/K] for constant volume *V* [m^3^], which is the same as for null work transfer interaction W=0 [J], ${\left(\partial S/\partial U\right)}_{W=0}$ [1/K]. At present, it is known that $S_{E,M,A}=S_{E,M,A}\left(N_{M,A}\right)$ [€] and $S_{E,M,B}=S_{E,M,B}\left(N_{M,B}\right)$ [€], and total derivatives could be used. However, for the future case, the analog of volume in Economics, the merchandise economic volume $V_{E,M}$ [?], can be proposed, and in that case, it will be $S_{E,M,A}=S_{E,M,A}\left(N_{M,A},V_{E,M,A}\right)$ [€] and $S_{E,M,B}=S_{E,M,B}\left(N_{M,B},V_{E,M,B}\right)$ [€], with an economic process at constant merchandise economic volume $V_{E,M}$ [?] being the one for which the merchandise wealth transfer interactions are null, $W_{M}=0$ [U]; the notation ${\left(\partial S_{E,M}/\partial N_{M}\right)}_{W_{M}=0}$ [€/U] was used; and the partial derivatives of the merchandise economic entropy were considered instead of its total derivatives.

### 3.6. Relevant Results of the New Proposed Approach

In addition to allow the absolute merchandise economic temperature (or the absolute merchandise unit price) to be defined, the new approach allows us to obtain other relevant results, which are reported in what follows.

From Figure 2, it is clear that if $S_{E,M,C}$ [€] reaches its maximum in the merchandise economic thermal equilibrium conditions, in these conditions it must be $d^{2}\left(S_{E,M,C}\right)/dr^{2}<0$ [€]. Using Equation (21), and taking into consideration Equations (18a) and (18b), it can be obtained that

(28) $$\frac{d^{2}\left(S_{E,M,C}\right)}{dr^{2}}={\left[\left(\frac{\partial^{2}S_{E,M,A}}{\partial N_{M,A}^{2}}\right)+\left(\frac{\partial^{2}S_{E,M,B}}{\partial N_{M,B}^{2}}\right)\right]}_{\delta W_{M}=\delta N_{M,g}=N_{M,W}=0}N_{M}^{2}\;\;[€]$$

and to be $d^{2}\left(S_{E,M,C}\right)/dr^{2}<0$ [€], if systems *A* and *B* are identical, it is required that under merchandise economic thermal equilibrium conditions, both systems *A* and *B*, as well as composite system *C*, satisfy

(29) $$\begin{array}{ll} {\left(\frac{\partial^{2}S_{E,M}}{\partial N_{M}^{2}}\right)}_{\delta W_{M}=\delta N_{M,g}=N_{M,W}=0}=\\ -\frac{1}{T_{E,M}^{2}}{\left(\frac{\partial T_{E.M}}{\partial N_{M}}\right)}_{\delta W_{M}=\delta N_{M,g}=N_{M,W}=0}<0 & \;\;[€/U^{2}] \end{array}$$

and thus we forcibly have

(30) $${\left(\frac{\partial T_{E,M}}{\partial N_{M}}\right)}_{\delta W_{M}=\delta N_{M,g}=N_{M,W}=0}>0\;\;[1/€]$$

that is, the merchandise economic temperature increases if the number of merchandise units in the economic system increases under merchandise economic thermal equilibrium conditions.

One major result, stemming from theoretical considerations, is that the merchandise economic temperature increases (the merchandise unit price decreases) as the number of traded merchandise units in the economic system increases. Thus, the theoretical result states that scarcity (small number of merchandise units) of tradable merchandise units dictates a higher merchandise unit price and that abundance (high number of merchandise units) of tradable merchandise units dictates a lower unit price, no matter the merchandise economic temperatures (the merchandise unit prices) of the merchandise units in the economic systems exchanging traded merchandise units with the economic system under consideration.

It is noted from ref. [24] that a positive change in the number of merchandise units in the economic system $\left(dN_{M}>0\right)$ [U] causes a tendency for the ‘escape’ of these merchandise units from the system $\left(dT_{E,M}>0\right)$ [U/€], $\left(dp_{M}<0\right)$ [€/U], that is, the economic system naturally tends to moderate the increase in $N_{M}$ [U] by enhancing its tendency to give up merchandise units. This natural reaction to a perturbation by favoring the re-establishment of the initial state, by moderating the perturbation, is a characteristic manifestation of the stability of equilibrium.

From Equation (30), it is obtained that the merchandise economic temperature (the inverse of the merchandise unit price) can only be positive, as no evidence exists that if the number of merchandise units in an economic system increases, their merchandise economic temperature decreases (their merchandise unit price increases). Given the way in which the absolute merchandise economic temperature is defined by Equation (23) and its positivity, it is the *absolute merchandise economic temperature*.

**Figure 3:**
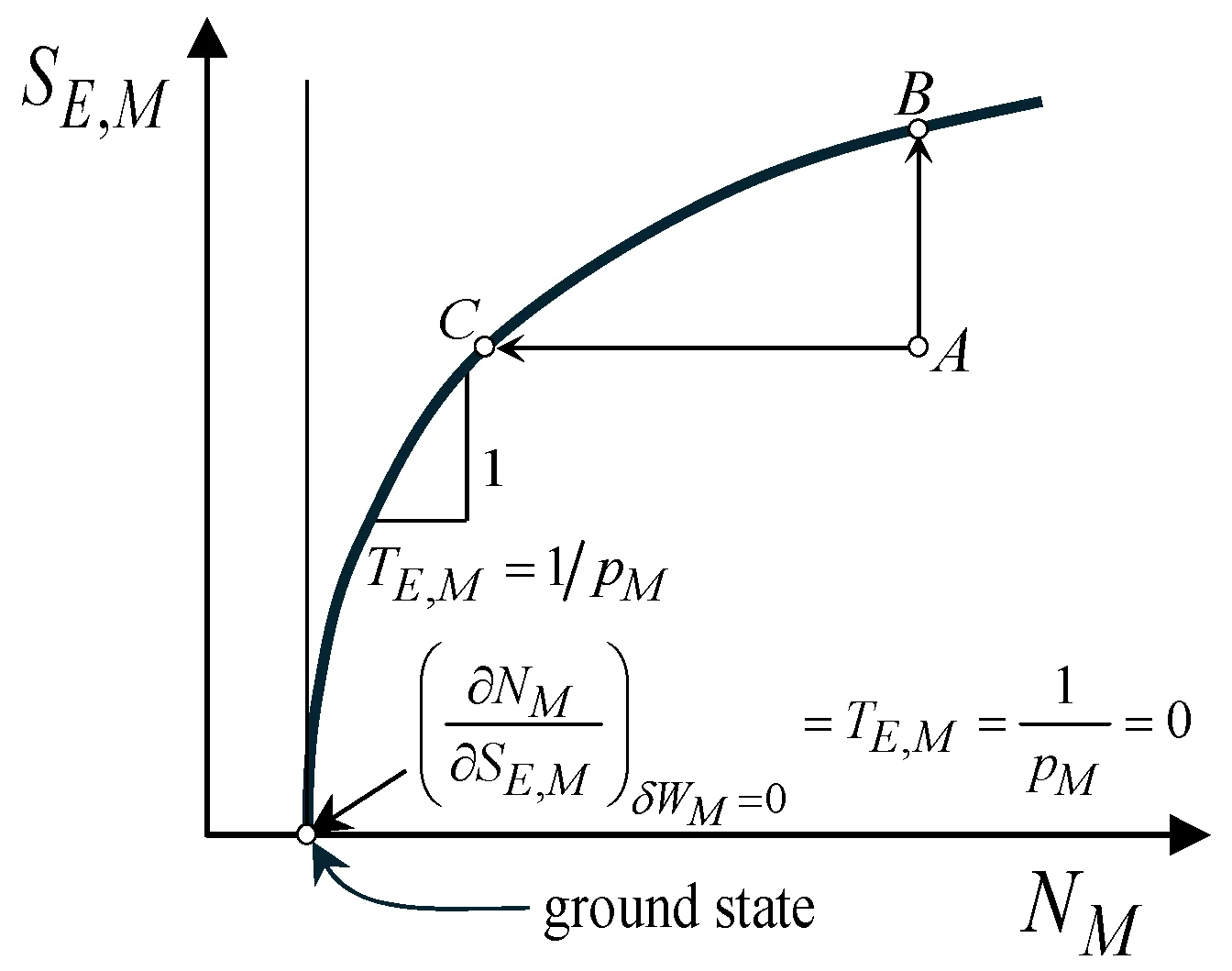
Schematic representation of the relationship between $S_{E,M}$ [€] and $N_{M}$ [U].

Given the previous analysis and developments, the way in which $S_{E,M}$ [€] and $N_{M}$ [U] are related is schematically represented in Figure 3.

The curve in Figure 3 corresponds to a set of stable merchandise economic thermal equilibrium states, for which the merchandise economic temperature is defined. Below that curve is a region of possible metastable (merchandise economic thermal non-equilibrium) states, for which the merchandise economic temperature is not defined. Additionally, looking at Figure 3, starting from point *A* and moving under constant $N_{M}=N_{M,A}$ [U], point *B* represents a limit, in this case, the maximum allowable $S_{E,M}$ [€] for a constant $N_{M}=N_{M,A}$ [U]. Analogously, starting from point *A* and moving under constant $S_{E,M}=S_{E,M,A}$ [€], point *C* represents a limit, in this case, the minimum allowable $N_{M}$ [U] for a constant $S_{E,M}=S_{E,M,A}$ [€]. The curve in Figure 3 can thus be seen as the set of stable merchandise economic thermal equilibrium states, with those equilibrium states corresponding to the maximum merchandise economic entropy and the minimum number of merchandise units. An economic system under the considered conditions (isolated, and with $\delta W_{M}=\delta N_{M,g}=N_{M,W}=0$ [U]) in a merchandise economic thermal non-equilibrium metastable state spontaneously evolves towards an equilibrium state represented by the curve in Figure 3, towards the maximum merchandise economic entropy, and towards the minimum number of merchandise units.

From Equations (23), (29), and (31) and Figure 3, it can thus be concluded that

(a) If a small $\Delta N_{M}$ [U] is associated with a large $\Delta S_{E,M}$ [€], that means that $T_{E,M}$ [U/€] is low and thus that $p_{M}$[€/U] is high;
(b) If a large $\Delta N_{M}$[U] is associated with a small $\Delta S_{E,M}$[€], that means that $T_{E,M}$[U/€] is high and thus that $p_{M}$ [€/U] is low;
(c) If $T_{E,M}\to 0$[U/€] and thus $p_{M}\to +\infty$[€/U], the slope of the curve in Figure 3 tends to infinity, which happens for $S_{E,M}\to 0$[€]. This corresponds to stating that if $T_{E,M}\to 0$[U/€], $S_{E,M}\to 0$ [€], that is, the merchandise economic entropy (the merchandise financial value) of an economic system whose merchandise economic temperature tends to zero (whose merchandise unit price tends to infinity) tends to zero. This is the statement of the Third Law of Economics.
(d) The state for which $T_{E,M}=0$[U/€] corresponds to $S_{E,M}=0$[€], even for $N_{M}>0\;\;\left(N_{M}\geq 0\right)$ [U], which is thus a *ground state* [24].

**Figure 4:**
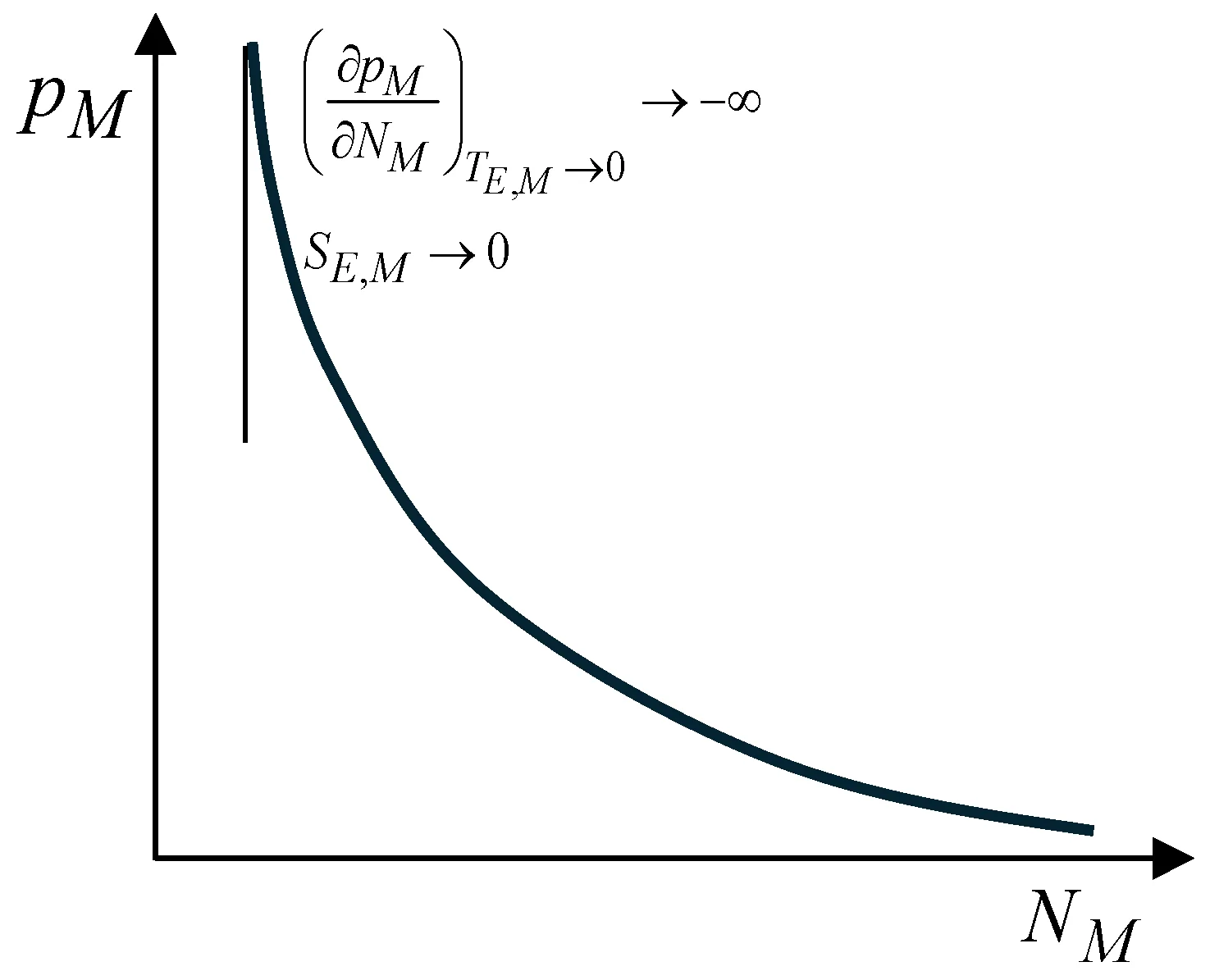
The intrinsic demand curve as a characteristic of the economic system itself.

If the curve in Figure 3 is represented differently, representing the inverse of the absolute merchandise economic temperature (the absolute merchandise unit price (the inverse of the slope of the curve in Figure 3, as given by Equation (26))) as a function of the number of tradable merchandise units $N_{M}$ [U] in the economic system, that leads to the *intrinsic demand curve*, as graphically illustrated in Figure 4 [29]. From Figure 3, it is clear that for $N_{M}\to +\infty$ [U], $\left(1/T_{E,M}\right)\to +0$ [€/U], that is, $p_{M}\to +0$ [€/U], and for the smallest values of $N_{M}$ [U], $\left(1/T_{E,M}\right)\to +\infty$ [€/U], that is, $p_{M}\to +\infty$ [€/U]. It is noted that Equation (30) can be rewritten as ${\left(\partial p_{M}/\partial N_{M}\right)}_{\delta W_{M}=\delta N_{M,g}=N_{M,W}=0}<0$ [€/U^2^], which is the mathematical ground for the negative slope of the intrinsic demand curve in Figure 4.

The curve in Figure 4 is a characteristic of the merchandise economic thermal equilibrium of the economic system itself and not a representation of the merchandise economic thermal equilibrium of the economic system under analysis with the neighboring economic systems with which it exchanges traded merchandise units. This is another relevant result, stating not only that the demand curve is dictated by the neighboring economic systems exchanging traded merchandise units with the economic system under analysis but also that the economic system under analysis has itself its own (characteristic) intrinsic demand curve. The condition that for $T_{E,M}\to 0$ [U/€] ($p_{M}\to +\infty$[€/U]) $S_{E,M}\to 0$ [€] corresponds to the upper-left region of the curve in Figure 4.

### 3.7. Concluding Step of the New Proposed Approach

The differential of the merchandise economic entropy property can be written as

(31) $$dS_{E,M}={\left(\frac{\partial S_{E,M}}{\partial N_{M}}\right)}_{\begin{array}{l} \delta W_{M}=\delta N_{M,g}=N_{M,W}=0\\ \mathrm{merchandise}\;\mathrm{economic}\\ \mathrm{thermal}\;\mathrm{equilibrium} \end{array}}dN_{M}\;\;[€]$$

which, considering Equation (23), can be rewritten as

(32) $$dS_{E,M}={\left(\frac{dN_{M}}{T_{E,M}}\right)}_{\begin{array}{l} \delta W_{M}=\delta N_{M,g}=N_{M,W}=0\\ \mathrm{merchandise}\;\mathrm{economic}\\ \mathrm{thermal}\;\mathrm{equilibrium} \end{array}}\;\;[€]$$

As for $\delta W_{M}=\delta N_{M,g}=0$ [U], the differential form of the merchandise units balance Equation (2) gives that $dN_{M}=\delta M$ [U] and $N_{M,W}=0$ [U], Equation (32) can be rewritten as

(33) $$dS_{E,M}={\left(\frac{\delta M}{T_{E,M}}\right)}_{\begin{array}{l} \mathrm{merchandise}\;\mathrm{economic}\\ \mathrm{thermal}\;\mathrm{equilibrium} \end{array}}\;\;[€]$$

As the previous results were obtained for an economic system in merchandise economic thermal equilibrium, for $\delta W_{M}=\delta N_{M,g}=N_{M,W}=0$ [U], under these conditions, it can be said that the economic system is in internal equilibrium, in which only reversible processes may occur, and Equation (33) can be written as

(34) $$dS_{E,M}\equiv {\left(\frac{\delta M}{T_{E,M}}\right)}_{\mathrm{int}\;\mathrm{rev}}\;\;[€]$$

the same result as obtained following the previously proposed approach [19]. This is the mathematical expression in the form of a differential equation defining the merchandise economic entropy.

It is noted that in most of previous processes considered, a number of zero merchandise wealth units in the economic system has been prescribed, that is, $N_{M,W}=0$ [U]. However, the same results could have been obtained if it were considered that it can be $N_{M,W}\neq 0$ [U], but in that case, just the number of tradable merchandise units $N_{M,M}$ [U] in the economic system must be considered instead of the total number of merchandise units $N_{M}$ [U] in the economic system.

### 3.8. On the Merchandise Economic Entropy Generation

The merchandise economic entropy is defined mathematically through Equation (34), with the involved derivative being for economically reversible processes in the economic system. For a general economic process, the change in merchandise economic entropy is greater than that associated with the exchanged traded merchandise units δM [U] for a reversible process $dS_{E,M}={\left(\delta M/T_{E,M}\right)}_{\mathrm{int}\;\mathrm{rev}}={\left(\delta M\cdot p_{M}\right)}_{\mathrm{int}\;\mathrm{rev}}$ [€]. If irreversible processes occur in the economic system, the change in the merchandise economic entropy of the system is greater than that associated with only the traded merchandise transfer interactions. This is obvious for δM≥0 [U] and an algebraic consequence for δM<0 [U]. In general terms, it can be written that

(35) $$dS_{E,M}\geq \frac{\delta M}{T_{E,M}}=\delta M\cdot p_{M}\;\;[€]$$

The equal sign in the inequality applies to internally reversible processes.

The positive of the difference in the terms involved in Equation (35) is defined as *merchandise economic entropy generation*:

(36) $$\delta S_{E,M,g}\equiv dS_{E,M}-\frac{\delta M}{T_{E,M}}=dS_{E,M}-\delta M\cdot p_{M}\geq 0\;\;[€]$$

This is the best indicator of the possible and impossible economic processes, as for the possible economic processes, it is forcibly $\delta S_{E,M,g}\geq 0$ [€] (positive, or in the reversible limit null, merchandise financial value generation). In the limiting situation of economically reversible processes, it is $\delta S_{E,M,g}=0$ [€] (null merchandise financial value generation).

Section 3.9 follows Section 2.6 of the work by Costa [20] closely.

### 3.9. Merchandise Economic Entropy Balance Equation

Written in the same form as usual in Engineering Thermodynamics [26], the merchandise economic entropy balance equation states that

time rate of changeof merchandiseeconomic entropyin the system︸in the system=net flow rate of tradedmerchandise economicentropy entering thesystem︸through the system's boundary+net merchandiseeconomic entropygeneration ratein the system︸in the system [€/s]

With the involved variables, the merchandise economic entropy balance equation can be written as

(37) $$\frac{dS_{E,M}}{dt}=\sum_{j=1}^{N_{Mj}}\frac{{\dot{M}}_{j}}{T_{E,j}}+{\dot{S}}_{E,M,g}\;[€/s]$$

In Thermodynamics, the work flow rate $\dot{W}$ [J/s] can be seen as a heat flow rate crossing the system’s boundary at infinite temperature: from the energy transfer viewpoint, it is the work flow rate transfer interaction $\dot{W}$ [J/s], and from the entropy viewpoint, it corresponds to the null entropy transfer interaction flow rate ${\left(\dot{W}/T\right)}_{T\to \infty }=0$ [J/(s.K)]. Similarly, the merchandise wealth flow rate ${\dot{W}}_{M}$ [U/s] can be seen as a traded merchandise flow rate crossing the economic system’s boundary at an infinite merchandise economic temperature (at a null merchandise unit price): from the merchandise transfer viewpoint, it is the merchandise wealth flow rate transfer interaction ${\dot{W}}_{M}$ [U/s], and from the merchandise economic entropy viewpoint, it corresponds to the null merchandise economic entropy flow rate ${\left({\dot{W}}_{M}/T_{E,M}\right)}_{T_{E,M}\to \infty }=0$ [€/s] [18,19,20].

Units of energy from the sun are available at null unit prices; they are available as merchandise wealth. If these units of energy are harvested and placed on the market, giving them non-zero-unit prices, merchandise wealth is then *converted* into traded merchandise. In principle, they will no longer by converted back into merchandise wealth (into units of energy available at null unit price).

In common language, it is common to say that the (true) wealth is not in the market; it is not to be purchased or sold; it has no price; or that, in the present context, it has a null merchandise unit price (it has an infinite merchandise economic temperature).

Multiplying Equation (37) by *dt* leads to the differential form of the merchandise economic entropy balance equation:

(38) $$dS_{E,M}=\sum_{j=1}^{N_{Mj}}\frac{\delta M_{j}}{T_{E,j}}+\delta S_{E,M,g}\;[€]$$

Monetary economic entropy balance equations can be similarly set [18,19,20]; however, they are not included here as they are not relevant for the purposes of this paper.

Beyond the concepts and results related to the Thermodynamics–Economics analogy, the time rate balance Equations (1) and (37), or their respective differential forms, Equations (2) and (38), are of major importance, allowing quantitative First and Second Law merchandise analyses of economic processes.

## 4. Conclusions

The Second Law of Thermodynamics is of recognized richness and, at the same time, strangeness, which motivates its invocation and consideration in many different fields and situations. This is also the case in Economics, with the Second Law bringing new insights, readings, and interpretations to subjects in Economics and to economic activity.

Entropy generation is a measure of the imperfection of thermodynamic processes; the challenge of science and engineering is for systems to operate at moderate levels of entropy generation. Similarly, economic entropy generation (financial value generation) can be considered a measure of the imperfection of economic processes; the challenge is for economic systems to operate at moderate levels of economic entropy generation.

A new approach to the Economics version of the Second Law is proposed and developed. It highlights how, from the observation of economic activity, and especially from the observation of normal trading operations, a statement of the Second Law can be set: the merchandise financial value of the traded merchandise units in an economic system cannot decrease. This defines the one-way behavior associated with the Second Law, thus also stating the possible and impossible economic processes. From the observation that the traded merchandise exchanges (trading operations) cease at equilibrium, the merchandise thermal equilibrium, for which the variable merchandise economic entropy (the merchandise financial value) is maximum, from that maximum condition, the absolute merchandise economic temperature (which is the inverse of the absolute merchandise unit price) is obtained.

If a function has a maximum, it is a concave function, and from that concavity condition, a relevant result is obtained, stating that if the merchandise economic temperature of the merchandise units in an economic system increases (if the merchandise unit price of the merchandise units in an economic system decreases), the number of tradable merchandise units in the economic system tends to increase, and vice versa. This main result allows another relevant result to be obtained: an economic system has its own characteristic *intrinsic demand curve*. Thus, that result teaches us that the merchandise demand is conditioned not only by the economic systems with which the economic system under analysis exchanges traded merchandise units but also by the economic system itself.

Analyzing the profile of the variation in the merchandise economic entropy of the economic system with the number of traded merchandise units in the economic system, the statement of the Third Law of Economics is obtained: the merchandise economic entropy (the merchandise financial value) of an economic system whose merchandise economic temperature tends to zero (whose merchandise unit price tends to infinity) also tends to zero.

Finally, returning to the equation defining the absolute merchandise economic temperature, the differential equation defining the merchandise economic entropy is obtained. The remaining steps are similar to the ones followed when proposing the previous approach to the Second Law of Economics.

Both Economics and Thermodynamics may benefit from the proposed new approach and, from the large set of concepts, developments, analyses, variables, relations, and results included in this work, from both scientific and pedagogical points of view.

## Figures and Tables

**Table 1:** Main Thermodynamics and Economics analogies from the previous works by Costa [18,19] and summarized in Section 2 of Costa [20].

Thermodynamics		Economics	
*m* [kg]	Mass in the system		
*E* [J]	Energy in the system		
		$N_{M}$ [U]	Number of merchandise units in the system
		$N_{m}$ [U]	Number of monetary units in the system
*U* [J]	Internal energy in the system	N [U]	Number of (merchandise and monetary) units in the system
*T* [K]	Temperature	$T_{E,M}$ [U/€]	Merchandise economic temperature
		$p_{M}$ [€/U]	Merchandise unit price
Q [J]	Heat transfer interaction	M [U]	Traded merchandise transfer interaction
$\dot{Q}$ [J/s]	Heat transfer flow rate	$\dot{M}$ [U/s]	Traded merchandise transfer flow rate
W [J]	Work transfer interaction	$W_{M}$ [U/s]	Merchandise wealth transfer interaction
$\dot{W}$ [J/s]	Work transfer flow rate	${\dot{W}}_{M}$ [U/s]	Merchandise wealth transfer flow rate
		$W_{m}$ [U/s]	Monetary wealth transfer interaction
		${\dot{W}}_{m}$ [U/s]	Monetary wealth transfer flow rate
		$N_{M,g}$ [U]	Merchandise units’ generation in the system
		${\dot{N}}_{M,g}$ [U/s]	Merchandise units’ generation rate in the system
		$N_{m,g}$ [U]	Monetary units’ generation in the system
		${\dot{N}}_{m,g}$ [U/s]	Monetary units’ generation rate in the system
$\oint \frac{\delta Q}{T}\leq 0$ [J/K]	Clausius inequality	$\oint \frac{\delta M}{T_{E,M}}\leq 0$ [€]	Merchandise Clausius inequality of Economics
$dS\equiv {\left(\frac{\delta Q}{T}\right)}_{\begin{array}{l} \mathrm{int}\\ \mathrm{rev} \end{array}}$ [J/K]	Differential equation defining entropy	$dS_{E,M}\equiv {\left(\frac{\delta M}{T_{E,M}}\right)}_{\begin{array}{l} \mathrm{int}\\ \mathrm{rev} \end{array}}$ [€]	Differential equation defining merchandise economic entropy
*S* [J/(kg·K)]	Entropy	$S_{E}$ [€]	Economic entropy
		$S_{E,M}$ [€]	Merchandise economic entropy
		$S_{E,m}$ [€]	Monetary economic entropy
$S_{g}$ [J/K]	Entropy generation in the system	$S_{E,g}$ [€]	Economic entropy generation in the system
		$S_{E,M,g}$ [€]	Merchandise economic entropy generation in the system
		$S_{E,m,g}$ [€]	Monetary economic entropy generation in the system
${\dot{S}}_{g}$ [U/s]	Entropy generation rate in the system	${\dot{S}}_{E,g}$ [€/s]	Economic entropy generation rate in the system
		${\dot{S}}_{E,M,g}$ [€/s]	Merchandise economic entropy generation rate in the system
		${\dot{S}}_{E,m,g}$ [€/s]	Monetary economic entropy generation rate in the system
$\eta_{C}=1-\frac{T_{2}}{T_{1}}$ [−]	Efficiency of the (reversible) Carnot engine	$\eta_{E,M,C}=1-\frac{T_{E,M,2}}{T_{E,M,1}}$ [−]	Efficiency of the (reversible) economic Carnot engine

## Data Availability

No numerical data were used in the paper.
